# A multi-ingredient food supplement slows age-dependent decline of mobility and influences gene expression in *C. elegans*

**DOI:** 10.1007/s10522-026-10463-8

**Published:** 2026-06-28

**Authors:** Chad Yanyatan, Karthik Mohanraj, Michael Fasseas, Sushmita Maitra, Ed Van Harmelen, Chris Saunter, David Weinkove

**Affiliations:** 1 Magnitude Biosciences Ltd., NETPark Plexus, Thomas Wright Way, Sedgefield, TS21 3FD UK; 2 Department of Biosciences, Durham University, Stockton Road, Durham, DH1 3LE UK; 3 Youth and Earth, Huckletree West-Mediaworks, 191 Wood Lane, London, W12 7FP UK

**Keywords:** Ageing, healthspan, *Caenorhabditis*, elegans, dietary supplementation, mobility decline, transcriptomics

## Abstract

The goal of developing interventions to slow ageing is not only lifespan extension but more importantly to increase healthspan, the period of life spent in active good health. Nutritional interventions have emerged as a potential strategy to maintain health with age. Testing these interventions for effects on human ageing would take several years and require large cohort sizes. We therefore employed *C. elegans* as a rapidly ageing model organism to investigate the effects of two commercially available nutrition-based products on ageing-related decline of mobility as an indication of healthspan. These products are multi-ingredient formulations comprising vitamins, minerals, antioxidants, amino acids and botanical extracts. They include compounds expected to positively influence ageing such as Dihydronicotinamide mononucleotide, NAD + booster, trans-resveratrol, taurine, pterostilbene and bioflavonoids. V14™ contains 40 + ingredients and AG1® contains 70 + ingredients with 5 additional probiotic strains. Mobility-based readouts over time were obtained using WormGazer™ imaging technology. Worms exposed to V14™ showed increased movement and speed with age compared to those exposed to AG1® and to a solvent control. To investigate the underlying mechanisms, transcriptomic profiling was performed on V14™ exposed worms, revealing modulation of pathways involved in metabolism and stress responses, processes associated with ageing. Collectively, these findings suggest that V14™ delays age-related decline in *C. elegans* and highlight the potential of targeted nutritional interventions to modulate pathways relevant to human ageing.

## Background

The average human lifespan has increased dramatically over the past century, resulting in increasingly aged populations across developed nations, a growing set of associated socioeconomic challenges (Christensen et al. 2009; Osareme et al. 2024) and increased risk of chronic disease (Niccoli and Partridge 2012). Whilst there are many conflicting definitions of the term healthspan (Masfiah et al 2025; Rattan 2026), there is a growing emphasis on extending the period of life spent in good health rather than absolute lifespan (Olshansky 2022; Osareme et al. 2024; Scott 2021). Ageing leads to the progressive deterioration of an organism’s functionality and makes it more vulnerable to death. It is now understood that ageing is, at least to some extent, modified by evolutionarily conserved genetic pathways and biochemical mechanisms, providing a basis for the development of targeted interventions (López-Otín et al. 2013).

Small molecules such as metformin, rapamycin, α-ketoglutarate and precursors of nicotinamide adenine dinucleotide have been extensively studied as candidates to slow ageing (Chin et al. 2014; Couteur and Barzilai 2022; Harrison et al. 2009; López-Otín et al. 2013). Alongside pharmacological approaches, nutritional interventions including polyphenols, fatty acids, and probiotics are gaining interest as newer strategies to support healthy ageing 2012 (Diwan and Sharma 2022; Gao et al. 2023; Pan et al. 2012; Wichansawakun and Buttar 2018; Pagliai et al. 2021). In parallel, the market for complex multi-ingredient nutritional supplements targeting health and longevity has expanded considerably (Clausen and Schlueter 2017; MacGregor et al. 2021). Rigorous preclinical evaluation of such products is therefore essential to substantiate efficacy claims. While human clinical trials remain the definitive standard, their significant cost and duration required for detecting delays in ageing make them out of reach for almost all new product development. Translatable and cost-effective model systems are therefore critical for the early-stage evaluation of interventions designed to extend healthspan. *Caenorhabditis elegans*, a free-living nematode maintained on an *Escherichia coli* diet in the laboratory, is a well-established model organism for ageing research (Lapierre and Hansen 2012; Markaki and Tavernarakis 2020). This worm is amenable to a wide array of genetic tools and resources while being a fast and cost-effective means to assess novel compounds or interventions for their effects on healthspan or lifespan (Weinkove and Zavagno 2021). Recently, use of the WormGazer™ imaging technology to monitor changes in movement with age has provided a rapid and automated means to measure multiple endpoints that are indicative of worm health during ageing (Jongsma et al. 2024; Zavagno et al. 2024). Monitoring *C. elegans* movement from early to mid-adulthood can rapidly identify interventions that slow ageing while simultaneously revealing any negative effects on health.

In this study, we combine the WormGazer™ Automated Healthspan assay, with a follow-up manual lifespan assay and a transcriptomic analysis to investigate the effects of V14™, a commercially available multi-ingredient supplement on *C. elegans* health during ageing. We compared V14™ to AG1®, another commercially available multi-ingredient powder for daily consumption. These products are comprised of vitamins, minerals, antioxidants, amino acids and botanical extracts. Both formulations comprise vitamins, minerals, antioxidants, amino acids and botanical extracts, with ingredients relevant to longevity pathways including NAD + precursors and bioflavonoids; V14™ provides 40 + and AG1® 70 + active ingredients, the latter incorporating additional probiotic strains (Tables 1 and 2). Each formulation is designed to target physiological pathways associated with enhanced human health. (V14 Longevity Reds, n.d.; What Is AG1?, n.d.) .

**Table 1:** V14™ ingredients and references from the literature

Ingredients	Literature demonstrating positive effects on human health	Literature demonstrating positive effects in *C. elegans*
Glycine	https://doi.org/10.1111/j.1479-8425.2007.00262.x https://doi.org/10.1111/j.1479-8425.2006.00193.x	https://pubmed.ncbi.nlm.nih.gov/30845140/
Magnesium-Malate	https://link.springer.com/article/10.1007/s12011-019-01663-0 https://pubmed.ncbi.nlm.nih.gov/29425476/	Not Available
Calcium AKG (Alpha-Ketoglutarate)	https://pubmed.ncbi.nlm.nih.gov/34847066/ https://pubmed.ncbi.nlm.nih.gov/37217632/	https://pubmed.ncbi.nlm.nih.gov/24828042/ https://pubmed.ncbi.nlm.nih.gov/37940787/
Taurine	https://pubmed.ncbi.nlm.nih.gov/29546641/ https://pubmed.ncbi.nlm.nih.gov/26781281/	https://pmc.ncbi.nlm.nih.gov/articles/PMC10630957/
Acetyl-L-Carnitine HCI	https://pubmed.ncbi.nlm.nih.gov/29076953/ https://pubmed.ncbi.nlm.nih.gov/12598816/	https://pubmed.ncbi.nlm.nih.gov/33290254/
NMNH (β-Dihydronicotinamide Mononucleotide)	https://www.sciencedirect.com/science/article/pii/S2161831323013595	https://pmc.ncbi.nlm.nih.gov/articles/PMC9039735/
Vitamin C (as Ascorbic Acid)	https://pubmed.ncbi.nlm.nih.gov/29099763/ https://pubmed.ncbi.nlm.nih.gov/23440782/	https://pubmed.ncbi.nlm.nih.gov/25346348/
Ginger	https://pmc.ncbi.nlm.nih.gov/articles/PMC5750786/ https://pubmed.ncbi.nlm.nih.gov/36364048/	https://pubmed.ncbi.nlm.nih.gov/36052763/
Fisetin	https://pubmed.ncbi.nlm.nih.gov/39384074/	https://pubmed.ncbi.nlm.nih.gov/36558979/
Pterostilbene	https://pubmed.ncbi.nlm.nih.gov/25132861/ https://pubmed.ncbi.nlm.nih.gov/18954071/ https://pmc.ncbi.nlm.nih.gov/articles/PMC4099343/	Not Available
Quercetin	https://pubmed.ncbi.nlm.nih.gov/37513932/ https://www.mdpi.com/2072-6643/8/3/167	https://pubmed.ncbi.nlm.nih.gov/19043800/
Trans-resveratrol	https://pmc.ncbi.nlm.nih.gov/articles/PMC6943596/ https://pmc.ncbi.nlm.nih.gov/articles/PMC7026982/	https://pubmed.ncbi.nlm.nih.gov/27190265/
Zinc (as Zinc Citrate)	https://pmc.ncbi.nlm.nih.gov/articles/PMC10539547/ https://pmc.ncbi.nlm.nih.gov/articles/PMC10874324/	https://pmc.ncbi.nlm.nih.gov/articles/PMC4831763/
Lithium microdose	https://www.nature.com/articles/s41586-025-09335-x https://pmc.ncbi.nlm.nih.gov/articles/PMC12030194/	https://pmc.ncbi.nlm.nih.gov/articles/PMC3151375/
Carotenoids (Lutein, Astaxanthin, Zeaxanthin)	https://www.frontiersin.org/journals/nutrition/articles/10.3389/fnut.2025.1522302/full https://pubmed.ncbi.nlm.nih.gov/36986186/	https://pubs.rsc.org/en/content/articlelanding/2025/fo/d4fo03490b https://pmc.ncbi.nlm.nih.gov/articles/PMC8750055/
Spermidine	https://pmc.ncbi.nlm.nih.gov/articles/PMC8612618/ https://pmc.ncbi.nlm.nih.gov/articles/PMC12245200/	https://pmc.ncbi.nlm.nih.gov/articles/PMC7521492/
Copper (as Copper Gluconate)	https://pmc.ncbi.nlm.nih.gov/articles/PMC8970836/ https://www.frontiersin.org/journals/molecular-biosciences/articles/10.3389/fmolb.2022.1065265/full	https://pubmed.ncbi.nlm.nih.gov/41575937/
Lithium (as Lithium Citrate)	https://pmc.ncbi.nlm.nih.gov/articles/PMC9925675/	https://pubmed.ncbi.nlm.nih.gov/17959600/ https://pubmed.ncbi.nlm.nih.gov/24398558/
B12 (as Methylcobalamin)	https://pubmed.ncbi.nlm.nih.gov/35532908/ https://pubmed.ncbi.nlm.nih.gov/32722436/	https://pmc.ncbi.nlm.nih.gov/articles/PMC8522492/
Vitamin K2 (as Menaquinone-7, MK-7)	https://pmc.ncbi.nlm.nih.gov/articles/PMC8483258/ https://www.frontiersin.org/journals/endocrinology/articles/10.3389/fendo.2025.1703116/full	https://pubmed.ncbi.nlm.nih.gov/35495953/
5-MTHF (5-Methyltetrahydrofolate)	https://pmc.ncbi.nlm.nih.gov/articles/PMC9380836/ https://pubmed.ncbi.nlm.nih.gov/39339754/	https://pmc.ncbi.nlm.nih.gov/articles/PMC8190293/
Selenium (as Sodium Selenite)	https://www.frontiersin.org/journals/nutrition/articles/10.3389/fnut.2023.1136458/full https://pubmed.ncbi.nlm.nih.gov/33884538/	https://pmc.ncbi.nlm.nih.gov/articles/PMC6499701/
Vitamin D3 (as Cholecalciferol)	https://www.mdpi.com/2072-6643/12/5/1248 https://pubmed.ncbi.nlm.nih.gov/40551736/	https://pubmed.ncbi.nlm.nih.gov/36001277/
L-Carnitine	https://pubmed.ncbi.nlm.nih.gov/34842765/ https://pubmed.ncbi.nlm.nih.gov/34959912/	https://pubmed.ncbi.nlm.nih.gov/33290254/
Carrot powder	https://pmc.ncbi.nlm.nih.gov/articles/PMC8065932/ https://pmc.ncbi.nlm.nih.gov/articles/PMC11606860/ https://www.tandfonline.com/doi/full/10.1080/10942912.2023.2301569#d1e595	https://pubmed.ncbi.nlm.nih.gov/38065964/
Beetroot powder	https://www.sciencedirect.com/science/article/pii/S2667031325002167 https://pubmed.ncbi.nlm.nih.gov/30400267/	https://pubmed.ncbi.nlm.nih.gov/35088214/ https://pubmed.ncbi.nlm.nih.gov/33924155/
Tomato powder	https://pubmed.ncbi.nlm.nih.gov/29317772/ https://pubmed.ncbi.nlm.nih.gov/35278075/	https://pmc.ncbi.nlm.nih.gov/articles/PMC5387417/
Montmorency Tart Cherry powder	https://pubmed.ncbi.nlm.nih.gov/35119142/ https://pmc.ncbi.nlm.nih.gov/articles/PMC10443385/	https://pmc.ncbi.nlm.nih.gov/articles/PMC7285199/
Pomegranate powder	https://pmc.ncbi.nlm.nih.gov/articles/PMC3678830/ https://www.mdpi.com/2072-6643/17/7/1235	https://pmc.ncbi.nlm.nih.gov/articles/PMC6764419/
Blueberry powder	https://pubmed.ncbi.nlm.nih.gov/36066009/ https://pubmed.ncbi.nlm.nih.gov/30999017/	https://pmc.ncbi.nlm.nih.gov/articles/PMC1413581/
Raspberry powder	https://pubmed.ncbi.nlm.nih.gov/33348685/ https://pubmed.ncbi.nlm.nih.gov/34501954/	https://pubmed.ncbi.nlm.nih.gov/32285078/
Strawberry powder	https://pubmed.ncbi.nlm.nih.gov/40199714/ https://pubmed.ncbi.nlm.nih.gov/27464461/	https://pubmed.ncbi.nlm.nih.gov/34628121/
Acai Berry powder	https://pubmed.ncbi.nlm.nih.gov/36839349/ https://www.mdpi.com/2076-3921/12/7/1443	https://pubmed.ncbi.nlm.nih.gov/26809379/
Plum powder	https://pubmed.ncbi.nlm.nih.gov/28422064/ https://pubmed.ncbi.nlm.nih.gov/34095361/ https://pmc.ncbi.nlm.nih.gov/articles/PMC9869099/	https://pubmed.ncbi.nlm.nih.gov/40350339/
Rhubarb powder	https://pmc.ncbi.nlm.nih.gov/articles/PMC7448319/ https://pmc.ncbi.nlm.nih.gov/articles/PMC12558105/	https://pubmed.ncbi.nlm.nih.gov/40086491/
Dragon Fruit powder	https://pubmed.ncbi.nlm.nih.gov/37087207/ https://www.mdpi.com/1420-3049/29/23/5676	https://pubmed.ncbi.nlm.nih.gov/36704792/
Grape Seed extract (4:1)	https://pubmed.ncbi.nlm.nih.gov/21802563/ https://pubmed.ncbi.nlm.nih.gov/41149258/ https://pmc.ncbi.nlm.nih.gov/articles/PMC7054588/	https://pubmed.ncbi.nlm.nih.gov/39682936/
Blackcurrant extract (4:1)	https://pubmed.ncbi.nlm.nih.gov/32460873/ https://pmc.ncbi.nlm.nih.gov/articles/PMC7267005/ https://pmc.ncbi.nlm.nih.gov/articles/PMC10563683/ https://pmc.ncbi.nlm.nih.gov/articles/PMC7056812/	https://www.sciopen.com/article/10.26599/FSHW.2025.9250893
Amla fruit extract (4:1)	https://pubmed.ncbi.nlm.nih.gov/31890983/ https://pubmed.ncbi.nlm.nih.gov/37128923/	https://journals.sagepub.com/doi/10.1177/1934578X1801301019
Acerola fruit extract (4:1)	https://pubmed.ncbi.nlm.nih.gov/30150795/ https://pubmed.ncbi.nlm.nih.gov/38396766/	Not Available
Cranberry extract (4:1)	https://pubmed.ncbi.nlm.nih.gov/37068952/ https://pubmed.ncbi.nlm.nih.gov/29046404/ https://pmc.ncbi.nlm.nih.gov/articles/PMC3823508/	https://pubmed.ncbi.nlm.nih.gov/22864793/
Bilberry fruit extract (4:1)	https://pmc.ncbi.nlm.nih.gov/articles/PMC7146147/ https://pubmed.ncbi.nlm.nih.gov/28617532/ https://www.ncbi.nlm.nih.gov/books/NBK92770/	https://pubmed.ncbi.nlm.nih.gov/32531930/
Bioflavonoids (Citrus extract lavonoids 30%)	https://pubmed.ncbi.nlm.nih.gov/30962863/ https://pubmed.ncbi.nlm.nih.gov/40904779/	https://pmc.ncbi.nlm.nih.gov/articles/PMC8001597/ https://pmc.ncbi.nlm.nih.gov/articles/PMC7723185/
Black Pepper (Piperine)	https://pmc.ncbi.nlm.nih.gov/articles/PMC8796742/ https://link.springer.com/chapter/10.1007/978-3-319-41334-1_8	https://pmc.ncbi.nlm.nih.gov/articles/PMC7237707/
Citric Acid	https://pmc.ncbi.nlm.nih.gov/articles/PMC10817003/ https://pmc.ncbi.nlm.nih.gov/articles/PMC2243251/	https://pubmed.ncbi.nlm.nih.gov/31745774/ https://pmc.ncbi.nlm.nih.gov/articles/PMC8672782/
Sorbitol	https://pubmed.ncbi.nlm.nih.gov/19047666/ https://pubmed.ncbi.nlm.nih.gov/28177738/	https://pmc.ncbi.nlm.nih.gov/articles/PMC4709409/
Stevia	https://pubmed.ncbi.nlm.nih.gov/39098209/ https://pmc.ncbi.nlm.nih.gov/articles/PMC8600158/	https://pubmed.ncbi.nlm.nih.gov/33567712/

**Table 2:** AG1® ingredients and references from the literature

Ingredients	Literature demonstrating positive effects	Literature demonstrating positive effects using *C. elegans*
Apple powder	https://pmc.ncbi.nlm.nih.gov/articles/PMC442131/ https://pmc.ncbi.nlm.nih.gov/articles/PMC4224039/ https://pmc.ncbi.nlm.nih.gov/articles/PMC10494637/	https://pubmed.ncbi.nlm.nih.gov/23878618/
Pea protein isolate	https://pmc.ncbi.nlm.nih.gov/articles/PMC12428665/ https://pmc.ncbi.nlm.nih.gov/articles/PMC4307635/ https://journal.lib.uoguelph.ca/index.php/surg/article/view/6111	Not available
Spirulina powder	https://pubmed.ncbi.nlm.nih.gov/32201580/ https://www.mdpi.com/2072-6643/16/5/642	https://pubmed.ncbi.nlm.nih.gov/32931825/
Lecithin (soy)	https://pmc.ncbi.nlm.nih.gov/articles/PMC11246377/ https://pmc.ncbi.nlm.nih.gov/articles/PMC5757297/	https://pubmed.ncbi.nlm.nih.gov/16122394/
Inulin (from chicory root)	https://pubmed.ncbi.nlm.nih.gov/39313030/ https://pubmed.ncbi.nlm.nih.gov/34555168/	https://pubmed.ncbi.nlm.nih.gov/40317421/
Magnesium bisglycinate	https://pubmed.ncbi.nlm.nih.gov/40918053/ https://pmc.ncbi.nlm.nih.gov/articles/PMC5637834/	Not available
Calcium salts of citric acid	https://pmc.ncbi.nlm.nih.gov/articles/PMC7935594/ https://pubmed.ncbi.nlm.nih.gov/31643038/	https://pubmed.ncbi.nlm.nih.gov/31745774/ https://pmc.ncbi.nlm.nih.gov/articles/PMC8672782/
Vitamin C (L-ascorbic acid)	https://pmc.ncbi.nlm.nih.gov/articles/PMC9925039/ https://www.frontiersin.org/journals/immunology/articles/10.3389/fimmu.2020.574029/full	https://pubmed.ncbi.nlm.nih.gov/25346348/
Citrus flavonoids	https://www.frontiersin.org/journals/nutrition/articles/10.3389/fnut.2025.1639901/full https://pubmed.ncbi.nlm.nih.gov/30962863/	https://pubmed.ncbi.nlm.nih.gov/31952185/ https://pubmed.ncbi.nlm.nih.gov/38897393/
Chlorella powder	https://pubmed.ncbi.nlm.nih.gov/23467073/ https://pmc.ncbi.nlm.nih.gov/articles/PMC8200412/ https://pubmed.ncbi.nlm.nih.gov/29037431/	https://pubmed.ncbi.nlm.nih.gov/38940020/
Wheatgrass powder	https://pmc.ncbi.nlm.nih.gov/articles/PMC11121291/	Not available
Alfalfa leaf powder	https://pmc.ncbi.nlm.nih.gov/articles/PMC8413032/ https://pmc.ncbi.nlm.nih.gov/articles/PMC4609025/	https://pubmed.ncbi.nlm.nih.gov/30489645/
Barley leaf powder	https://pmc.ncbi.nlm.nih.gov/articles/PMC5904770/ https://pubmed.ncbi.nlm.nih.gov/32318238/	https://pubmed.ncbi.nlm.nih.gov/34798183/
Broccoli powder	https://pmc.ncbi.nlm.nih.gov/articles/PMC4572790/ https://pmc.ncbi.nlm.nih.gov/articles/PMC10376324/ https://pmc.ncbi.nlm.nih.gov/articles/PMC12271217/	https://pubmed.ncbi.nlm.nih.gov/33471780/
Papaya fruit powder	https://pmc.ncbi.nlm.nih.gov/articles/PMC8066973/ https://pmc.ncbi.nlm.nih.gov/articles/PMC6682863/ https://pmc.ncbi.nlm.nih.gov/articles/PMC3984819/	Not available
Potassium salts of orthophosphoric acid	https://pmc.ncbi.nlm.nih.gov/articles/PMC11397259/	Not available
Beet root powder	https://pubmed.ncbi.nlm.nih.gov/30400267/ https://www.sciencedirect.com/science/article/pii/S2667031325002167?via%3Dihub	https://pubmed.ncbi.nlm.nih.gov/35088214/ https://pubmed.ncbi.nlm.nih.gov/33924155/
Antioxidant (citric acid)	https://pubmed.ncbi.nlm.nih.gov/24433072/ https://www.intechopen.com/chapters/62460	https://pubmed.ncbi.nlm.nih.gov/31745774/
*Lactobacillus rhamnosus* GG	https://www.nmi.health/lactobacillus-rhamnosus-gg-a-review-of-clinical-use-and-efficacy/ https://pmc.ncbi.nlm.nih.gov/articles/PMC4372813/	https://pubmed.ncbi.nlm.nih.gov/35127565/
*Lactobacillus acidophilus* NCFM	https://pubmed.ncbi.nlm.nih.gov/21436726/ https://pmc.ncbi.nlm.nih.gov/articles/PMC9668099/	https://pubmed.ncbi.nlm.nih.gov/22585961/
*Bifidobacterium lactis* HN019	https://pmc.ncbi.nlm.nih.gov/articles/PMC8712437/ https://pmc.ncbi.nlm.nih.gov/articles/PMC9409207/	https://pubmed.ncbi.nlm.nih.gov/23291976/
*Lactobacillus casei* LC-11	https://pmc.ncbi.nlm.nih.gov/articles/PMC7524140/ https://pubmed.ncbi.nlm.nih.gov/27169634/	Not available
*Lactobacillus* *plantarum* LP-115	https://pmc.ncbi.nlm.nih.gov/articles/PMC12532744/ https://pubmed.ncbi.nlm.nih.gov/15749636/ https://pubmed.ncbi.nlm.nih.gov/17897389/	Not available
Inositol	https://pubmed.ncbi.nlm.nih.gov/36703143/ https://pubmed.ncbi.nlm.nih.gov/35664247/	https://pubmed.ncbi.nlm.nih.gov/37047164/
Carrot root powder	https://www.mdpi.com/2223-7747/13/1/93 https://pmc.ncbi.nlm.nih.gov/articles/PMC6544626/	https://pubmed.ncbi.nlm.nih.gov/38065964/ https://pmc.ncbi.nlm.nih.gov/articles/PMC10709416/
Cocoa powder	https://pmc.ncbi.nlm.nih.gov/articles/PMC4696435/ https://pmc.ncbi.nlm.nih.gov/articles/PMC3488419/	https://journals.sagepub.com/doi/10.3233/NHA-200100
Licorice root powder	https://www.nccih.nih.gov/health/licorice-root https://pmc.ncbi.nlm.nih.gov/articles/PMC8703329/ https://pmc.ncbi.nlm.nih.gov/articles/PMC6657287/	https://pubmed.ncbi.nlm.nih.gov/31545123/
Acerola fruit extract	https://pubmed.ncbi.nlm.nih.gov/30150795/ https://pubmed.ncbi.nlm.nih.gov/38396766/	https://pubmed.ncbi.nlm.nih.gov/33672539/
Ginger root powder	https://pmc.ncbi.nlm.nih.gov/articles/PMC9654013/ https://pubmed.ncbi.nlm.nih.gov/31935866/	https://pubmed.ncbi.nlm.nih.gov/36052763/
Spinach leaf powder	https://pmc.ncbi.nlm.nih.gov/articles/PMC11845096/ https://pmc.ncbi.nlm.nih.gov/articles/PMC11593830/ https://pmc.ncbi.nlm.nih.gov/articles/PMC7916335/	https://pubmed.ncbi.nlm.nih.gov/40287100/
Bromelain	https://pubmed.ncbi.nlm.nih.gov/37157782/ https://pubmed.ncbi.nlm.nih.gov/38999808/	Not available
Calcium salts of orthophosphoric acid	https://pmc.ncbi.nlm.nih.gov/articles/PMC2884321/	Not available
Coenzyme Q-10 (ubiquinone)	https://www.ncbi.nlm.nih.gov/books/NBK531491/ https://www.academia.edu/107311182/Coenzyme_Q10_A_Review_of_Clinical_Use_and_Efficacy https://pmc.ncbi.nlm.nih.gov/articles/PMC8389239/	https://pubmed.ncbi.nlm.nih.gov/14706236/
Rose hip fruit extract	https://pubmed.ncbi.nlm.nih.gov/25834460/ https://pubmed.ncbi.nlm.nih.gov/18407528/	Not available
Choline L-bitartrate	https://pubmed.ncbi.nlm.nih.gov/36950691/ https://pmc.ncbi.nlm.nih.gov/articles/PMC6259877/	https://pubmed.ncbi.nlm.nih.gov/31379987/
Vitamin E (D-alpha-tocopheryl acid succinate)	https://pmc.ncbi.nlm.nih.gov/articles/PMC3997530/ https://pmc.ncbi.nlm.nih.gov/articles/PMC10374030/	https://pubmed.ncbi.nlm.nih.gov/3374177/
Alpha lipoic acid	https://pubmed.ncbi.nlm.nih.gov/36006850/ https://pmc.ncbi.nlm.nih.gov/articles/PMC11505271/ https://pmc.ncbi.nlm.nih.gov/articles/PMC6723188/	https://pubmed.ncbi.nlm.nih.gov/17156833/
Artichoke leaf extract	https://www.mdpi.com/2079-9284/11/3/69 https://pmc.ncbi.nlm.nih.gov/articles/PMC8398945/	https://pubmed.ncbi.nlm.nih.gov/35279029/
Sweetener (Steviol glycosides from stevia)	https://pubmed.ncbi.nlm.nih.gov/39098209/ https://pubmed.ncbi.nlm.nih.gov/27537496/	https://pubmed.ncbi.nlm.nih.gov/33567712/
Stabiliser (silicon dioxide)	https://efsa.onlinelibrary.wiley.com/doi/10.2903/j.efsa.2018.5088	Not Available
Zinc citrate	https://pmc.ncbi.nlm.nih.gov/articles/PMC10539547/ https://pmc.ncbi.nlm.nih.gov/articles/PMC10874324/	https://pmc.ncbi.nlm.nih.gov/articles/PMC4831763/
Pineapple fruit powder	https://pubmed.ncbi.nlm.nih.gov/38542694/ https://pubmed.ncbi.nlm.nih.gov/33233252/ https://pmc.ncbi.nlm.nih.gov/articles/PMC8028712/	https://papers.ssrn.com/sol3/papers.cfm?abstract_id=5702852
Reishi mushroom powder	https://www.ncbi.nlm.nih.gov/books/NBK92757/ https://scholarsarchive.byu.edu/facpub/1609/	https://pubmed.ncbi.nlm.nih.gov/34091442/
Shiitake mushroom powder	https://pubmed.ncbi.nlm.nih.gov/25866155/ https://pmc.ncbi.nlm.nih.gov/articles/PMC3199106/ https://pubmed.ncbi.nlm.nih.gov/11860402/	https://pubmed.ncbi.nlm.nih.gov/35041882/
Slippery elm bark powder	https://pubmed.ncbi.nlm.nih.gov/11860402/ https://pmc.ncbi.nlm.nih.gov/articles/PMC6065514/	Not available
Rutin	https://pmc.ncbi.nlm.nih.gov/articles/PMC10435270/ https://pubmed.ncbi.nlm.nih.gov/11083486/	https://pubmed.ncbi.nlm.nih.gov/33988888/
Astragalus root extract	https://www.mdpi.com/2673-8392/4/1/14	https://pmc.ncbi.nlm.nih.gov/articles/PMC6844597/
Rosemary leaf extract	https://pmc.ncbi.nlm.nih.gov/articles/PMC7491497/ https://pmc.ncbi.nlm.nih.gov/articles/PMC10045493/	https://pmc.ncbi.nlm.nih.gov/articles/PMC4988476/
Niacin (niacinamide)	https://pubmed.ncbi.nlm.nih.gov/41088896/ https://pubmed.ncbi.nlm.nih.gov/38671873/	https://pmc.ncbi.nlm.nih.gov/articles/PMC6982340/
Selenium enriched yeast	https://pubmed.ncbi.nlm.nih.gov/40244176/ https://pmc.ncbi.nlm.nih.gov/articles/PMC11872778/	Not available
Fucoidan extract from seaweed *Fucus vesiculosus*	https://pmc.ncbi.nlm.nih.gov/articles/PMC7866543/ https://pmc.ncbi.nlm.nih.gov/articles/PMC10399747/	https://pmc.ncbi.nlm.nih.gov/articles/PMC4584361/
Lycium berry fruit extract	https://www.mdpi.com/2673-4168/5/2/35 https://pmc.ncbi.nlm.nih.gov/articles/PMC5758351/	https://pmc.ncbi.nlm.nih.gov/articles/PMC8790518/
Dandelion extract	https://pmc.ncbi.nlm.nih.gov/articles/PMC9002813/ https://pmc.ncbi.nlm.nih.gov/articles/PMC5341965/	https://pubmed.ncbi.nlm.nih.gov/31912839/
Vitamin B6 (pyridoxal 5 ‘-phosphate)	https://www.sciencedirect.com/science/article/abs/pii/S0261561418301675 https://onlinelibrary.wiley.com/doi/10.1002/hup.2852	https://pubmed.ncbi.nlm.nih.gov/34011272/
Eleuthero root extract	https://pmc.ncbi.nlm.nih.gov/articles/PMC12195798/	https://pubmed.ncbi.nlm.nih.gov/18536978/
Pantothenic acid (D-pantothenate calcium)	https://pmc.ncbi.nlm.nih.gov/articles/PMC10770646/ https://pubmed.ncbi.nlm.nih.gov/31691401/	https://pmc.ncbi.nlm.nih.gov/articles/PMC12226095/
Burdock root extract	https://pubmed.ncbi.nlm.nih.gov/20981575/ https://pmc.ncbi.nlm.nih.gov/articles/PMC5658563/ https://pmc.ncbi.nlm.nih.gov/articles/PMC7686739/	https://www.sciencedirect.com/science/article/abs/pii/S0031942215300339?via%3Dihub
Niacin (nicotinic acid)	https://pubmed.ncbi.nlm.nih.gov/24391126/ https://pmc.ncbi.nlm.nih.gov/articles/PMC10420858/	https://pubmed.ncbi.nlm.nih.gov/31878234/
Vitamin B1 (thiamin hydrochloride)	https://pmc.ncbi.nlm.nih.gov/articles/PMC10568373/ https://pubmed.ncbi.nlm.nih.gov/40647310/	https://pmc.ncbi.nlm.nih.gov/articles/PMC9714793/
Hawthorn berry extract	https://pmc.ncbi.nlm.nih.gov/articles/PMC7047282/ https://pubmed.ncbi.nlm.nih.gov/19789403/	https://pubmed.ncbi.nlm.nih.gov/36172739/
Vitamin B2 (riboflavin)	https://pmc.ncbi.nlm.nih.gov/articles/PMC8401857/ https://www.nature.com/articles/s41522-024-00579-5	https://pmc.ncbi.nlm.nih.gov/articles/PMC8522492/
Sodium molybdate (molybdenum (VI))	https://pmc.ncbi.nlm.nih.gov/articles/PMC6837345/ https://pubmed.ncbi.nlm.nih.gov/15193982/ https://pubmed.ncbi.nlm.nih.gov/23906370/	https://pubmed.ncbi.nlm.nih.gov/36423681/
Bilberry fruit extract	https://pmc.ncbi.nlm.nih.gov/articles/PMC7146147/ https://pubmed.ncbi.nlm.nih.gov/28617532/ https://www.ncbi.nlm.nih.gov/books/NBK92770/	https://pubmed.ncbi.nlm.nih.gov/32531930/
Manganese bisglycinate	https://pmc.ncbi.nlm.nih.gov/articles/PMC12412596/ https://pmc.ncbi.nlm.nih.gov/articles/PMC6525788/	https://pubmed.ncbi.nlm.nih.gov/16545686/
Cupric gluconate	https://pmc.ncbi.nlm.nih.gov/articles/PMC9762617/ https://pmc.ncbi.nlm.nih.gov/articles/PMC8308383/	https://pubmed.ncbi.nlm.nih.gov/39726988/
Vitamin A (beta-carotene)	https://pmc.ncbi.nlm.nih.gov/articles/PMC3139236/ https://pmc.ncbi.nlm.nih.gov/articles/PMC10605009/	https://pubmed.ncbi.nlm.nih.gov/36304081/
Milk thistle seed extract	https://www.ncbi.nlm.nih.gov/books/NBK11896/ https://pmc.ncbi.nlm.nih.gov/articles/PMC9588316/ https://pmc.ncbi.nlm.nih.gov/articles/PMC3586829/	https://pubmed.ncbi.nlm.nih.gov/25613505/
Grape seed extract	https://pubmed.ncbi.nlm.nih.gov/21802563/ https://pubmed.ncbi.nlm.nih.gov/41149258/ https://pmc.ncbi.nlm.nih.gov/articles/PMC7054588/	https://pubmed.ncbi.nlm.nih.gov/39682936/
Folic acid (calcium-L-methylfolate)	https://pmc.ncbi.nlm.nih.gov/articles/PMC9380836/ https://pmc.ncbi.nlm.nih.gov/articles/PMC11081602/ https://pmc.ncbi.nlm.nih.gov/articles/PMC8235569/	https://pubmed.ncbi.nlm.nih.gov/26546011/
Biotin (D-biotin)	https://pmc.ncbi.nlm.nih.gov/articles/PMC5582478/ https://pmc.ncbi.nlm.nih.gov/articles/PMC5129763/	https://pubmed.ncbi.nlm.nih.gov/26158509/
Chromium picolinate	https://pmc.ncbi.nlm.nih.gov/articles/PMC7192664/ https://pubmed.ncbi.nlm.nih.gov/32804855/ https://pmc.ncbi.nlm.nih.gov/articles/PMC8997060/	Not available
Vitamin B12 (methylcobalamin)	https://pubmed.ncbi.nlm.nih.gov/35532908/ https://pubmed.ncbi.nlm.nih.gov/32722436/	https://pubmed.ncbi.nlm.nih.gov/34592146/ https://pubmed.ncbi.nlm.nih.gov/38247478/ https://pubmed.ncbi.nlm.nih.gov/35032420/
Vitamin K (menaquinone-7)	https://pmc.ncbi.nlm.nih.gov/articles/PMC8483258/ https://www.frontiersin.org/journals/endocrinology/articles/10.3389/fendo.2025.1703116/full	https://pubmed.ncbi.nlm.nih.gov/35495953/

Here, we present evidence that V14™ delays age-related mobility decline in *C. elegans* and provides a strong preclinical rationale for its further evaluation as a nutritional strategy to support healthy ageing. The transcriptional response to V14™ provides intriguing avenues for future research.

## Methods

### Strains and culture of* C. elegans*

*Escherichia coli* strain OP50 and *C. elegans* strains were obtained from the Caenorhabditis Genetics Center (CGC) which is funded by the NIH Office of Research Infrastructure Programs (P40OD010440). The strains used were N2, and SS104 *(glp-4(bn2). glp-4(bn2)* worms are temperature-sensitive sterile and are used in the WormGazer™ and lifespan assays at the restrictive temperature of 24 °C to prevent progeny interfering with the data **(**Zavagno et al. 2024). The strain was maintained at a permissive temperature of 15 °C. For consistency and convenient timing, both strains were maintained at the same temperature.

### Agar plate preparation

Petri dishes were filled with defined media (DM) in which peptone found in the standard nematode growth medium (Brenner 1974) is replaced with defined amino acids and trace metals to minimise a batch-to-batch variation in peptone as previously described (Virk et al. 2016) with vitamin B12 added (Maynard et al. 2018).

Even though doses in nematodes and humans cannot be compared due to different routes of entry and pharmacokinetics, we were conscious that V14™ and AG1® were given in different doses (15 g and 13 g respectively), so the experiments were designed with a final concentration of 1.56 mg/ml for V14™, and 1.25 mg/ml for AG1®. We diluted the products in DMSO as we found that they were not sufficiently soluble in water. Even in DMSO, a centrifugation was needed to remove insoluble debris. A 312.5 mg/ml master stock solution (100×) was made in DMSO for V14™, and a 250 mg/ml master stock solution (100×) was made in DMSO for AG1®. The master stock solutions were then centrifuged at 6000 rpm for 10 min to pellet suspended particles. Supernatants were collected and centrifuged at 15000 rpm for 10 min to pellet any remaining suspended particles. The supernatants were collected and further diluted with DMSO to yield stock solutions at 156 mg/ml for V14™ and at 125 mg/ml for AG1®. 1.5 ml of each stock solution was added to 150 ml of 1 × agar solution, to yield a final test concentrations of 1.56 mg/ml for V14™, and 1.25 mg/ml for AG1®. For control plates, 1.5 ml of DMSO was added to 150 ml of agar media.

Petri dishes for imaging were poured with DM agar 3 days before the worms were added. On the next day, Petri dishes were seeded with 100 µL of an overnight culture of *E. coli* OP50. Petri dishes were stored at 20 °C with controlled humidity until 24 h after bacterial seeding, when they were transferred to an incubator at 24 °C.

### *C. elegans *growth and development assessment

To ensure that there was no acute or developmental toxicity from the preparations, an assay called a DoseFinder (Zavagno et al. 2024) was performed by treating the wild type N2 worms with 5 mg/ml, 2.5 mg/ml and 1.25 mg/ml of each product. The plates with these concentrations of the products were then seeded with *E. coli* and, after 24 h, bacterial growth and lawn were assessed visually. Four N2 L4 larval stage worms were added to each plate at 24 °C. Progeny on-set and development were assessed qualitatively on Days 3 and 5, time points at which second-generation animals appear. Bacterial lawn consumption was also assessed qualitatively as an additional readout of worm growth and development. There were no effects on bacterial growth or *C. elegans* growth and development, and thus the doses chosen for the subsequent assays as described above were appropriate.

### Healthspan assays using WormGazer™

To prepare for experiments on the WormGazer™, gravid adult worms were placed onto 9-cm Petri dishes to lay eggs 4 days before being placed on the machine. The mothers were removed after 48 h, and 24 h later, these 9-cm Petri dishes were shifted to 24 °C to ensure large numbers of L4s are present (in case of N2) and to induce sterility in the case of SS104 *glp-4(bn2)* worms. On the following day, 12 L4 larval stage worms were selected and picked onto each 3.5-cm Petri dishes. Plates were then loaded into the machines and left undisturbed for the duration of the experiment.

Plates were continuously monitored using WormGazer™ as described (Zavagno et al. 2024). After imaging for seven days, any plates that failed a quality control inspection were censored, and omitted from data analysis, for example if they showed the presence of bacterial or fungal contamination, or the worms burrowed into the agar.

### Manual carry-on lifespan assays

Immediately after the completion of the WormGazer™ assay, all the worms were manually transferred to new 6-cm plates containing respective compounds for manual lifespan scoring. Plates were scored manually until mortality of the entire worm population. Worms were prodded with a platinum pick every 48 h to check for movement before being marked as dead. Worms were transferred on days 7 and 14 to new plates without the compounds. Worms that had bagged, burst vulvas or crawled off the plate were censored. Kaplan–Meier curves were generated using GraphPad Prism.

### RNA-seq analysis

Worms were exposed to V14™ on agar plates at a concentration of 1.56 mg/ml as described above for 3 or 6 days. A minimum of 60 worms per replicate were collected, with four biological replicates per condition. Worms were washed thoroughly with M9 buffer and preserved in RNA-stabilizing solution until use. RNA extraction and sequencing was performed by Novogene (UK). GO enrichment analysis was performed using Panther (https://geneontology.org/).

### Statistical analysis

For the data from the WormGazer™ experiments, the shading on the time-series curves is 1 standard error of the mean across the curves for each Petri dish within the condition. The error bars on the bar charts are ± 1 standard error of the mean across the measurements for each dish within the condition. When comparing two conditions on a bar chart, the difference between the means is calculated, and the standard error on this is calculated with reference to the standard error on each individual measure, using Gaussian error statistics. The significance thresholds are then set with reference to the difference expressed in terms of its standard error. A difference of less than 1.64 standard errors is marked as not significant (ns). A difference between 1.64 and 2.33 standard errors is marked as one star (*), corresponding to p < 0.05 on a one-sided test. A difference between 2.33 and 2.83 standard errors is marked as two stars (**), corresponding to p < 0.01 on a one-sided test. A difference greater than 2.83 standard errors is marked as three stars (***) corresponding to p < 0.002 on a one-sided test. For the lifespan assays, statistical significance was determined using Log Rank and Wilcoxon tests of the Kaplan–Meier survival model.

## Results

### Exposure to V14™ delays age-dependent mobility decline

**Fig. 1:**
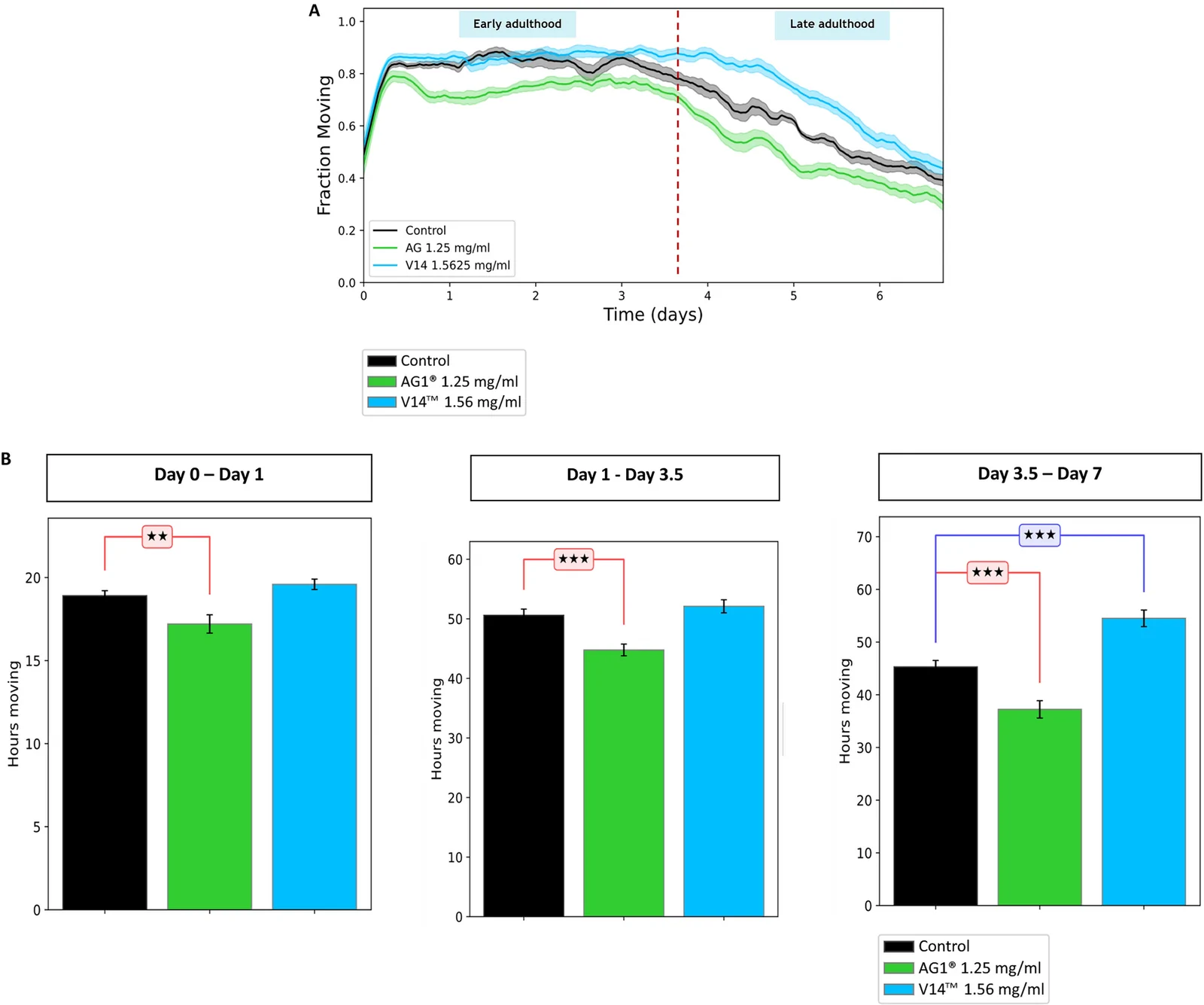
Healthspan assay of worms exposed to 1.56 mg/ml V14™, 1.25 mg/ml AG1® or DMSO control, using WormGazer. **A** The fraction moving graph shows the proportion of worms moving during the imaging window with SEM shading and **B** the area under the curve integration for hours moving on days 0–1, 1–3, and 3.5–7. n ≥ 108, 144 and 142 worms, 9–12 3.5 cm Petri dishes per condition, ** = p < 0.01, *** = p < 0.002, one-tailed test

The multi-endpoint analysis of movement using WormGazer™ revealed that, for both biological repeats, exposure to V14™ had a positive impact on the mobility of the worms during ageing (day 3–7) in comparison to AG1® and the control. Exposure to AG1® on the other hand, had a negative effect compared to the control. Specifically, V14™ displayed increase in the fraction of worms moving during late adulthood from Day 3.5 to Day 5.5 (Fig. 1A). This effect was not seen during young adulthood (Day 0.5 to Day 3.5). AG1® decreased the fraction of worms moving throughout the whole experiment (Day 0–7). Integrating the area under the curve from the fraction of worms moving over time provides the hours moved by the worms (Fig. 1B). Here, V14™ treatment led to a statistically significant increase in distance moved during late adulthood (after Day 3.5) when compared to AG1® and the control, while the effect of AG1® was negative when compared to the control.

**Fig. 2:**
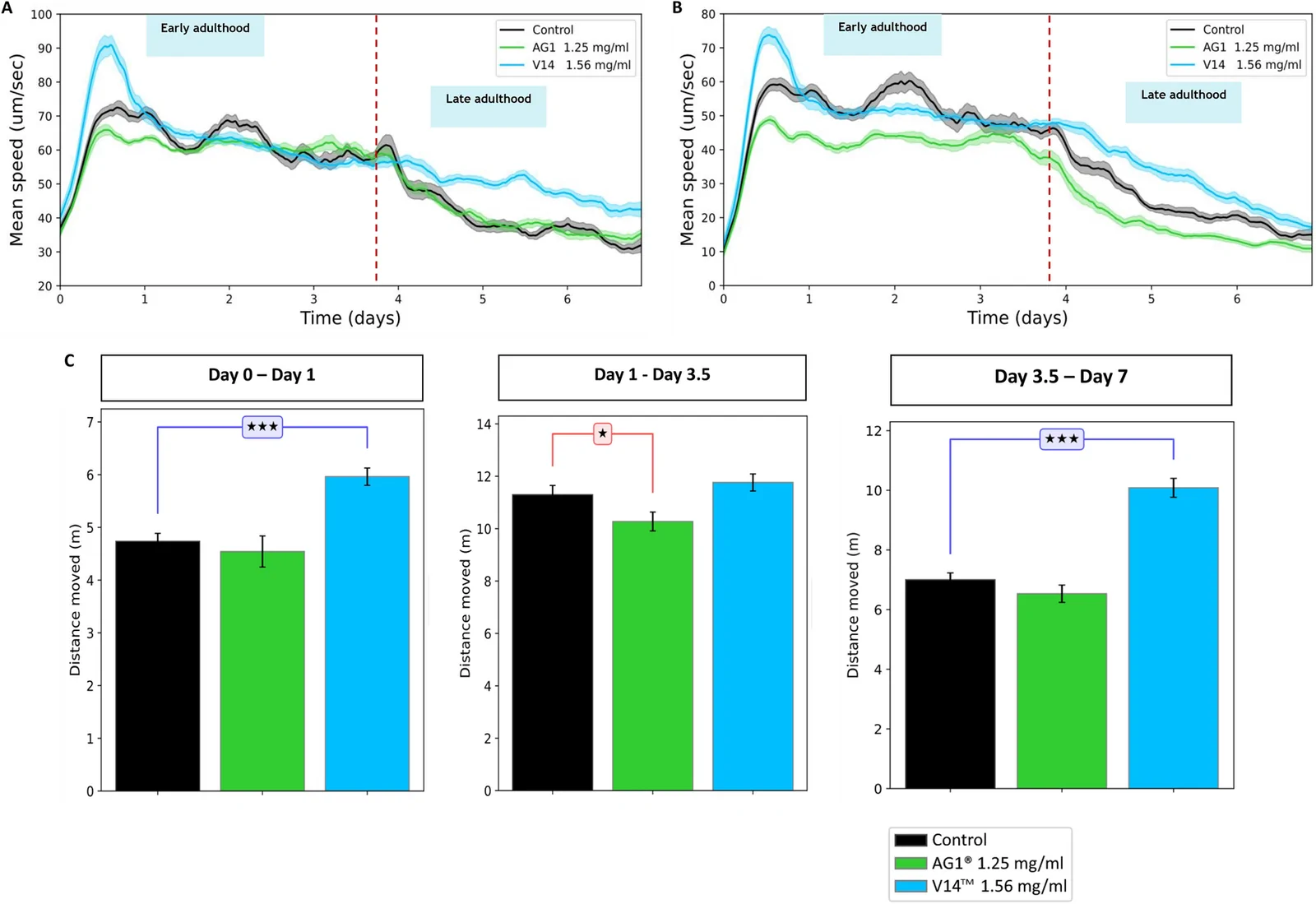
Healthspan assay of worms exposed to 1.56 mg/ml V14™, 1.25 mg/ml AG1® or DMSO control, using WormGazer. **A** The mean speed of moving worms, **B** the mean speed of all worms, and **C** the area under the curve integration for days 0–1, 1–3,4 and 3,5–7 for the distance moved (**B**). n ≥ 108, 144 and 142 worms, 9–12 Petri dishes per condition, * = p < 0.05, *** = p < 0.002, one-tailed test

With respect to speed of the worms that are moving, V14™ treatment had a positive effect very early (before Day 1) and then during late adulthood (after Day 3.5) when compared to control and AG1®. Exposure to AG1® reduced the speed of the moving worms, compared to the control (Fig. 2A). A similar effect was also observed for the speed of all worms (moving and not moving) (Fig. 2B). Integrating the area under the curve over time provides the distance moved by the worms (Fig. 2C). In both biological repeats, V14™ demonstrated a statistically significant increase in the distance moved by the worms before Day 1 and after Day 3.5, when compared to the control. AG1® caused a statistically significant decrease in the distance moved between Day 1 and Day 3.5 in both repeats however the negative effect after Day 3.5 was only statistically significant in one of the experiments.

### Exposure to V14™ or AG1® does not affect lifespan

**Fig. 3:**
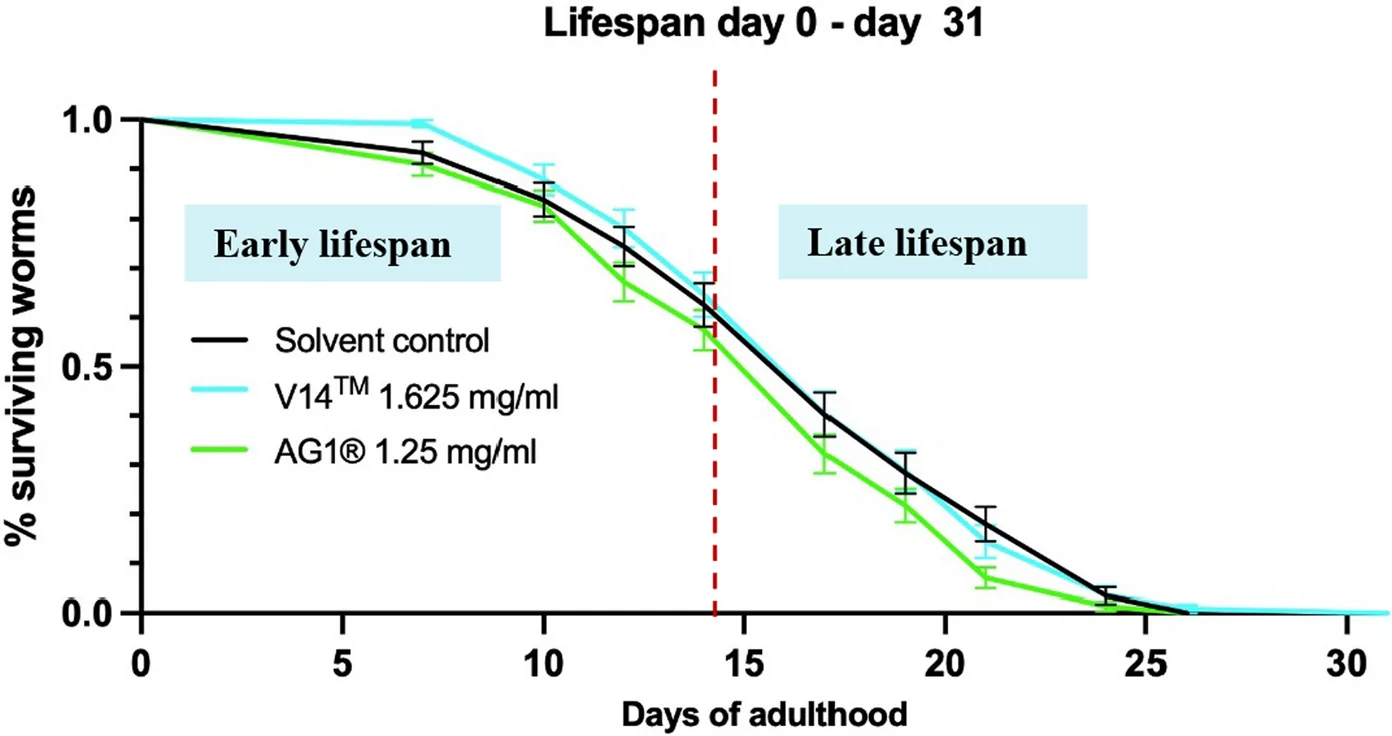
Lifespan assay of *glp-4* mutant worms exposed to 1.56 mg/ml V14™, 1.25 mg/ml AG1® or DMSO control, using WormGazer™ and manual picking after Day 7. The survival curve shows the proportion of worms alive at each timepoint. n ≥ 109, 131, 144 worms accordingly, 5–6 6 cm Petri dishes per condition, ** = p < 0.01, *** = p < 0.002, one-tailed test

Following the automated healthspan assay, the worms were subjected to manual lifespan assays. Across two experiments there was not a reproducible effect on lifespan from exposure to V14™ or to AG1® (Fig. 3).

### V14™ induces a transcriptional response in *C. elegans*

**Fig. 4:**
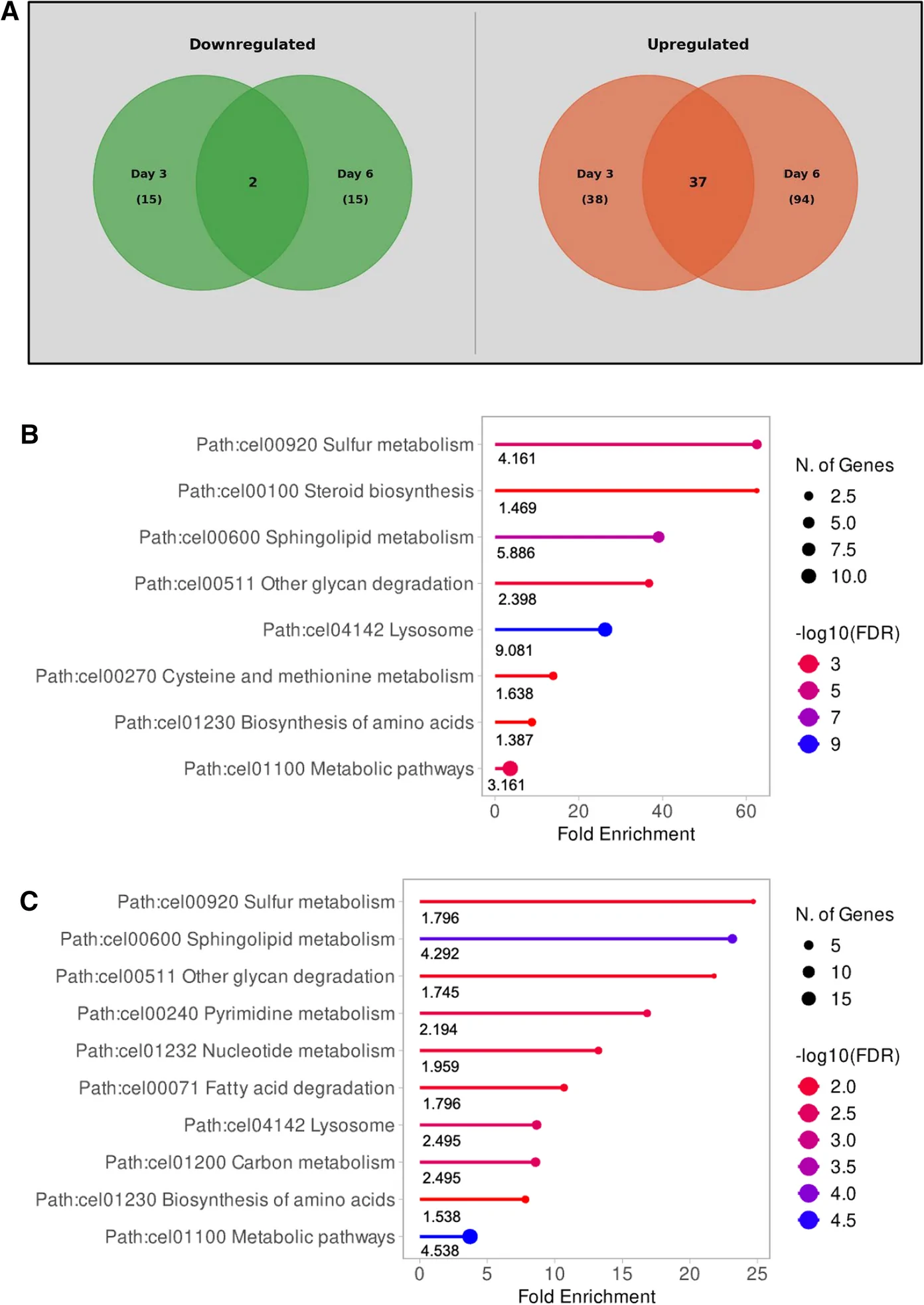
Transcriptomic analysis of *glp-4* mutant worms exposed for 3 or 6 days to 1.56 mg/ml V14™, or DMSO control, using RNA-seq. **A** Venn diagrams showing differentially expressed genes (FDR P < 0.05), **B** GO term analysis of upregulated genes on Day 3, **C** GO term analysis of upregulated genes on Day 6. The values on B and C are numerical representations of the − log_10_ (FDR) numbers rounded to 3 decimal places. 4B and 4 C were created using ShinyGO 0.80 (Ge et al*.* 2020)

A transcriptional analysis using RNA-seq was performed on worms exposed to 1.56 mg/mL of V14™ for 3 and 6 days. The results show a statistically significant transcriptional response at both time points (padj < 0.05, Fig. 4). On Day 3, 17 genes were downregulated and 75 were upregulated. By Day 6, 17 genes were downregulated and 131 were upregulated. Notably, 2 downregulated genes and 37 upregulated genes were shared between both time points (Fig. 4A). A GO enrichment analysis with an FDR P < 0.05, showed that there were no significant results by biological process with respect to the upregulated genes at either time-point. However, on Day 3 and on Day 6, the genes that were upregulated were enriched for several biological processes as shown in Fig. 4B and C.

Transcriptomic analysis revealed a predominantly upregulated response to V14™ treatment at both timepoints, with progressive upregulation of xenobiotic detoxification and sphingolipid metabolism genes from Day 3 to Day 6. Genes involved in hydrogen sulfide catabolism and mitochondrial respiratory chain function were consistently regulated in directions associated with reduced metabolic burden and maintained mitochondrial integrity. Notably, pathways associated with ageing including insulin/IGF-1 signalling, TOR, AMPK, and autophagy were not transcriptionally engaged. The mechanistic basis of the observed mobility improvement remains to be established through targeted functional studies.

## Discussion

As food supplements increasingly move beyond the prevention of nutrient deficiencies to target ageing and age-related diseases, there is a growing need for rapid, efficient screening methods to evaluate their effects in experimental systems in which ageing can be observed The aim of this study was to evaluate the effects of V14™ on age-related mobility parameters in *C. elegans*. V14™ contains a large number of ingredients that individually are believed, but not yet shown, to exert a positive effect on human longevity. Because mobility is a complex phenotype that depends on many physiological processes like muscle and neuronal functional coordination, it is an excellent readout for the health status of *C. elegans*. Furthermore, tracking the decline of mobility over time provides a dynamic measure of the ageing process. We hypothesised that if any constituents of V14™, individually or through nutrient synergy, confer protection against age-related mobility decline, this would be detectable in, *C. elegans* using the WormGazer™ technology. The study included another product AG1® that also contains ingredients with proposed beneficial effects on human health.

AG1® has already been shown in preclinical studies to act as a symbiotic leading to significantly increased total SCFA production, acetate, propionate, and other metabolic byproducts of fermentation in a transplanted human colonic microbiota (Kirby et al. 2023). In an in vitro experiment it was shown that supplementation with AG1® might also stimulate protective effects on gastrointestinal tract permeability, possibly as a result of increased SCFA production (Sapp et al. 2024). In a randomized, double-blind, placebo-controlled clinical trial, AG1® supplementation was shown to enrich beneficial taxa in the microbiome and could improve digestive quality of life (La Monica et al. 2024).

Although AG1® has been proposed to provide multiple benefits including reduced tiredness and fatigue, increased mental performance and cognitive function, and to support digestion and immunity, there are no claims specifically regarding longevity. V14™ on the other hand, was designed to improve longevity by comprising ingredients that are thought to target the hallmarks of ageing. The composition of both V14™ and AG1® are presented in Tables 1 and 2 together with available peer-reviewed publications supporting ageing-related effects of each ingredient. While both supplements share a core group of ingredients, V14™ contains a unique suite of compounds absent in AG1®. Notably, NMNH has been shown to suppress glycolysis (Liu et al. 2021), a finding that aligns with the metabolic changes suggested by our transcriptional analysis. Furthermore, trans-resveratrol has been demonstrated to significantly decrease total cholesterol in human clinical trials (Cao et al. 2022) and plays a critical role in regulating lipid and glucose metabolism (Zhou et al. 2022). While identifying the specific bioactive responsible for the observed phenotypic improvements is challenging in a multi-component formula, it is plausible that NMNH, trans-resveratrol, or the synergy of other unique ingredients in V14™ drive these positive effects.

It has been demonstrated, using *C. elegans*, that extended lifespan does not necessarily correspond to improved healthspan. Bansal et al., (2015) showed that the long-lived *C. elegans* mutant strains *daf-2(e1370), eat-2(ad1113), ife-2(ok306)*, and *clk-1(qm30)* increased the proportion of time spent in a frail state. In another study, where muscle strength was measured as an indicator of health in the long-lived *C. elegans* mutant strains *eat-2(ad1116), daf-2(e1370), daf-2(e1368)* and *glp-1(e2141)*, healthspan was not reduced but rather subjected to temporal scaling (Statzer et al. 2022). A recent review concluded that dietary restriction (DR)-induced lifespan extension in *C. elegans*, also extends healthspan (Loo et al. 2023). Whether or not different concentrations of V14™ could also impact lifespan remains to be seen but the hypothesis remains, that interventions to improve health in ageing humans, will have a significant effect on quality of life and may also extend lifespan by delaying the onset of age-related diseases (Kaeberlein et al. 2015; Milman and Barzilai 2023).

The GO enrichment analysis of the RNAseq did not reveal any statistically significant enrichment in the genes downregulated on Day 3 or Day 6 (FDR P < 0.05). However, the upregulated genes on Day 3 were enriched in GO terms involved in the catabolism of sphingolipids, membrane lipids and glucosylceramide (Fig. 4B, C). Sphingolipids are complex lipids that are abundant in the cell membranes affecting their structure and fluidity, regulating various biological processes, including signal transduction, cell differentiation, apoptosis, and autophagy (Li et al. 2025). Glucosylceramides are a class of sphingolipids that have been shown to play a role in *C. elegans* lifespan. For instance, C22 glucosylceramide is critical *for glp-1* mediated lifespan extension while compromising glucosylceramide production results in a dramatic reduction in lifespan of worms on glucose (Xatse and Olsen 2023). On Day 6, the upregulated genes were enriched in GO terms involved in response to stress, biotic stimuli, symbionts, other organisms and immunity. It is well known that such responses are closely related to longevity in *C. elegans*. The regulation of immunity by both the PMK-1 pathway and the DAF-2/DAF-16 pathway is a key determinant of lifespan (Troemel et al. 2006). The transcriptional changes are consistent with the worms boosting lipid remodelling in early adulthood (day 3), remodelling their membrane composition to maintain cellular homeostasis, before shifting toward mounting immune and stress defences during late adulthood (day 6).

Whether or not exposure to V14™ leads to increased catabolism of sphingolipids that in turn modulate processes that delay declines in mobility will require more experimentation but the small overlap between the DEGs of the two timepoints suggests that *C. elegans* responds differently to the presence of V14™ at different ages. Indeed, it is not possible to say whether these genes are differentially regulated as a direct result of V14™ or whether they changed because the worms are more metabolically young, as seen from the movement data. Further experimentation will be needed to investigate whether these results indicate a transcriptomic shift toward a slower ageing state. Taken together, the results from our experiments demonstrate that supplementing *C. elegans* with V14™ leads to improvement of mobility linked to increased healthspan without affecting lifespan. Moreover, it can be hypothesized that this effect is not due to increased nutrient supply, but rather to modulation of metabolic pathways in response to one or more of the ingredients comprising V14™.

## Conclusions, limitations and future directions

This study provides the first evidence that the multi-ingredient nutritional supplement V14™ slows age-related mobility decline in *C. elegans* and results in transcriptional changes that can guide further study.

Nevertheless, it should be mentioned that there are some limitations in the current study. While *C. elegans* provides a well-established and efficient model for studying conserved ageing pathways, differences in physiology, pharmacology and metabolism mean that results cannot be directly translated to humans. V14™ needs to be tested across a wide range of doses to define the optimal effective range. Individual ingredients need to be tested separately and in combinations to assess how they contribute to any slowing of mobility decline. And even after that analysis the dosing and exposure conditions in this model do not directly correspond to human use. The transcription changes observed require further investigation and only show correlation. To establish functional links with ageing, genetics experiments would be required.

In addition, healthspan assessment was primarily based on mobility, which represents one important aspect of functional ageing. Other functional assays could be included such as stress responses and behavioural assays. Nonetheless, the observed effects on movement and associated molecular pathways provide supportive, hypothesis-generating evidence for further investigation. In conclusion, V14™ delays age-related functional decline in *C. elegans* and engages conserved biological pathways with direct relevance to human ageing.

## Data Availability

All of the raw and processed data gathered from the RNAseq analysis will be uploaded to NCBI public database and also be available upon request.
